# Modes of Viroid Transmission

**DOI:** 10.3390/cells11040719

**Published:** 2022-02-18

**Authors:** Ahmed Hadidi, Liying Sun, John W. Randles

**Affiliations:** 1 U.S. Department of Agriculture, Agricultural Research Service, Beltsville, MD 20705, USA; 2 State Key Laboratory of Crop Stress Biology for Arid Areas, College of Plant Protection, Northwest A&F University, Yangling, Xianyang 712100, China; sunliying@nwafu.edu.cn; 3 School of Agriculture, Food and Wine, The University of Adelaide, Adelaide, SA 5005, Australia; john.randles@adelaide.edu.au

**Keywords:** viroids, modes of transmission, seed, pollen, vectors, insects, ascomycete fungi, mycoviroids (fungal viroids), cDNA clones or transcripts, disease control

## Abstract

Studies on the ways in which viroids are transmitted are important for understanding their epidemiology and for developing effective control measures for viroid diseases. Viroids may be spread via vegetative propagules, mechanical damage, seed, pollen, or biological vectors. Vegetative propagation is the most prevalent mode of spread at the global, national and local level while further dissemination can readily occur by mechanical transmission through crop handling with viroid-contaminated hands or pruning and harvesting tools. The current knowledge of seed and pollen transmission of viroids in different crops is described. Biological vectors shown to transmit viroids include certain insects, parasitic plants, and goats. Under laboratory conditions, viroids were also shown to replicate in and be transmitted by phytopathogenic ascomycete fungi; therefore, fungi possibly serve as biological vectors of viroids in nature. The term “mycoviroids or fungal viroids” has been introduced in order to denote these viroids. Experimentally, known sequence variants of viroids can be transmitted as recombinant infectious cDNA clones or transcripts. In this review, we endeavor to provide a comprehensive overview of the modes of viroid transmission under both natural and experimental situations. A special focus is the key findings which can be applied to the control of viroid diseases.

## 1. Introduction

Many diseases of unknown etiology with symptoms similar to those caused by plant viruses but for which no virions could be found were described during the middle half of the twentieth century. In 1971, Diener demonstrated that the causal agent of potato spindle disease, first described in the early 1920’s, is a small, highly structured, covalently closed circular RNA known as potato-spindle-tuber viroid (PSTVd) [1,2]. In 1972, Semancik and Wallace reported similar findings for the causal agent of citrus exocortis disease [3]. For such an unconventional agent the term viroid, suggested by Diener, was adopted in 1972 [4]. Viroids replicate autonomously, do not encode proteins, and use pre-existing host-cell RNA polymerase and processing enzymes for replication and pathogenesis [5,6]. According to the International Committee on Taxonomy of Viruses (ICTV) in 2020 and 2021, viroids are divided into two families: *Pospiviroidae* and *Avsunviroidae* including eight genera and thirty-three species [7]. In addition, based on sequence properties and/or autonomous replication, several viroids are classified as unassigned members of three genera [8]. Thus, the unassigned viroids in the genus *Apscaviroid* are apple-fruit-crinkle viroid (AFCVd) and grapevine-latent viroid (GLVd), in the genus *Coleviroid* are coleus-blumei viroid 5 (CbVd-5) and coleus-blumei viroid 6 (CbVd-6), and in the genus *Pospiviroid* is portulaca-latent viroid (PoLVd). Recently, two novel viroids have been described, apple-chlorotic-fruit-spot viroid (ACFSVd), a tentative new viroid in the genus *Apscaviroid* [9,10], and coleus-blumei viroid 7 (CbVd-7), a tentative new member of the genus *Coleviroid* [11].

We define viroid transmission as the process by which viroids spread between hosts, either of the same or different species. The natural host range of viroids includes vegetables, field and ornamental crops, fruit and palm trees, and grapevine [5,6]. Recently, an experimental test of the susceptibility of three phytopathogenic ascomycete fungi to seven viroids showed that some of these fungi could support the replication of certain viroids [12]. Identifying the modes of transmission of viroids such as asexual vegetative propagation, mechanical damage, sexual transmission via seed or pollen, or by vectors to new hosts are important for determining viroid-disease epidemiology, and for developing appropriate cultural control measures.

## 2. Vegetative Transmission

As they are systemic pathogens, viroids are transmitted where vegetative means of propagation are used. Apart from the monocotyledonous palms which host CCCVd and CTiVd and which do not have vegetative propagules, all other hosts subject to propagation by grafting, budding, cuttings, bulbs, tubers and other methods will carry viroid infection to the next planting. Somaclonal propagation that was developed for oil palm plantations is expected to be a route for CCCVd to be distributed via vegetatively propagated ramets.

## 3. Mechanical Transmission of Viroids

Mechanical transmission of viroids may occur by viroid-infected sap or nucleic acids or may occur by other means such as viroid-contaminated farming tools and agricultural and horticultural practices in which viroid-contaminated instruments are used. The dilution endpoint of between 10^−2^ and 10^−3^ for potato-leaf extracts [13] illustrates the high specific infectivity of PSTVd.

### 3.1. Viroid Genera of the Family Pospiviroidae

Genus *Pospiviroid*: PSTVd is mechanically transmitted by normal cultivation activities [14] and can be readily disseminated in potato fields by contact of healthy vines with contaminated cultivating and hilling tools and equipment [15]. Mechanical transmission of CEVd with contaminated budding tools from citron to citron can be very efficient [16]. The successful mechanical transmission of CEVd was also reported in commercial clementine and lemon as well as in commercial plantations subjected to standard agronomic practices [17]. CSVd is mechanically transmitted either by stem slashing with a viroid-contaminated razor blade [18,19] or by stem injury with viroid-contaminated-needle pricking [20]. CSVd can be transmitted by contaminated cutting tools [21]. Transmission in chrysanthemums by plant-to-plant contact also occurs [18]. Root contact has been reported to transmit the viroid [22]. IrVd-1 and PoLVd are transmitted mechanically; however, the efficiency of the mechanical inoculation of PoLVd may be low [23].

Genus *Hostuviroid*: for HSVd in hops, the primary mode of viroid transmission is through mechanical means [24]. Once the viroid becomes established in a hop planting, it is easily transmitted by workers, cutting tools, and equipment during cultural activities. DLVd is transmitted by mechanical inoculation; infected dahlia plants are asymptomatic [25].

Genus *Apscaviroid*: ASSVd may spread naturally from infected trees to uninfected neighboring trees by root grafting [26,27]. ASSVd is also transmitted mechanically by razor slashing of apple seedlings with viroid RNA preparation [28], and by using viroid-contaminated pruning tools, to both lignified stems and green shoots of apple trees and seedlings [29]. ADFVd is mechanically transmitted by stem slashing with razor blades contaminated with purified viroid RNA [30]. The apple isolate of AFCVd is transmitted mechanically by razor blade slashing of seedlings with a partially purified viroid preparation [31]. The mechanical transmission of the hop isolate is associated with farm operations that lead to mechanical injury [31]. PBCVd can be mechanically transmitted by stem-slash inoculation with a razor blade that was previously immersed in nucleic-acid preparations from infected tissue; these preparations may be mechanically inoculated to cucumber leaves [32].

Genus *Cocadviroid*: CCCVd and CTiVd are transmitted under experimental conditions to coconut and other palm seedlings by high-pressure injection and razor-blade slashing with nucleic-acid inoculum [33,34]. CBCVd and HLVd are transmitted in citrus orchards and hop gardens, respectively, by vegetative propagation, and mechanically by contaminated tools or machinery [17].

Genus *Coleviroid*: Coleus plants are vegetatively propagated with no active monitoring for viroid infection, which is often asymptomatic [11]. CbVd-1 can be transmitted to *Coleus blumei* and *Ocimum sanctum* by mechanical wounding [35].

### 3.2. Viroid Genera of the Family Avsunviroidae

Genus *Avsunviroid*: ASBVd is transmitted by mechanical means such as viroid-contaminated pruning and propagation tools [36,37]. Root grafting between avocado trees may be another means of transmission [38,39].

Genus *Pelamoviroid*: PLMVd in the peach is transmitted mechanically by slashing infected plants and then healthy ones [40] or by high-pressure injection with the viroid purified from infected tissue [41]. The presence of PLMVd in almond and pear trees neighboring infected peaches in Tunisia has been attributed to mechanical transmission by contaminated tools [42]. CChMVd is transmitted by the mechanical inoculation of crude extracts or total-nucleic-acid preparations from infected tissue [43].

Genus *Elaviroid*: ELVd has been mechanically transmitted by cutting tools [44].

## 4. Seed and Pollen Transmission

A number of viroids are known to be transmitted through seeds and/or pollen (Table 1). These modes of transmission may play a key role in the epidemiology of viroid diseases as epidemic outbreaks are dependent on the primary viroid inoculum brought in by viroid-infected seed at the beginning of the growing season and/or pollen at a later time. In conjunction with secondary viroid spread by mechanical and/or vector transmission, the introduction of viroids to new areas may lead to the development of viroid-disease epidemics. Moreover, international seed and pollen trade and exchange are considered one of the important contributing factors to the emergence of viroids and their diseases. There are several factors that influence the rate of seed and/or pollen transmission. These may include, but may not be limited to, plant species and cultivar, viroid variant, environmental conditions, and time of infection. Effective phytosanitary measures to control movement of pollen and seed are needed for these viroids.

PSTVd may have spread among potato germplasm collections world-wide in infected true seed [63]; it is pollen transmitted in tomato [70] and potato [61,69]. There is no evidence of seed transmission of CEVd in citrus species. However, in *Impatiens* and *Verbena* spp., relatively high but variable seed-transmission rates for the viroid were reported [50], which explains the occurrence and prevalence of CEVd in these hosts. There is evidence that TCDVd and CLVd are seed-transmitted in petunia and tomato [51]. In general, HSVd is not transmitted through seeds; however, seed transmission may play a role in the survival of HSVd in certain hosts such as grapevine [56]. ASSVd is seed-borne in apple [46], and seedlings germinated from ASSVd-positive apple seeds demonstrated a 7.7% infection rate [29]. ACFSVd is seed-transmitted from symptomatic apple fruit; seedlings that germinated from infected apple seeds showed an infection rate of 2.8% [10]. Transmission of GYSVd-1 via infected seeds in eight grapevine varieties has been demonstrated [56,57]. A low rate of CCCVd transmission to coconut palms by pollen and seeds was reported [49]. HLVd transmission by pollen or seeds has been reported to be low [58,59]. CbVd-1 is also seed-transmissible with the infection rate ranging from 0% to 100% depending on cultivars [47]. It was also reported that CbVd-5 and CbVd-6 can be transmitted via seeds [48]. *Coleviroid* infections are predominantly asymptomatic and they do not pose serious economic or agricultural threats [11]. ASBVd is transmitted in seeds and transmission rates of 86–100% have been observed in seeds from asymptomatic carrier trees, but rates are much lower in seeds from symptomatic trees (0–5.5%) [45]. ELVd is seed-transmitted with an efficiency of approximately 20% [44,55]. PLMVd in peach plants is pollen-borne and -transmitted [68]. Thus, healthy plants that were experimentally pollinated with the viroid-infected pollen resulted in infection with PLMVd at a rate dependent on the peach cultivar; after six years, PLMVd was detected in five cultivars [68].

## 5. Vector Transmission

A vector is defined by Merriam-Webster Dictionary as “an organism that transmits a pathogen from one organism or source to another”. An alternative definition of vector is: “an agent (such as plasmid or virus) that contains or carries modified genetic material (such as recombinant DNA) and can be used to introduce exogenous genes into the genome of an organism”. Recombinant-DNA technology was developed by Paul Berg in 1972 [71] for which he received the Nobel Prize in Chemistry in 1980. 

### 5.1. Transmission by Insects, Parasitic Plants and Goats

Viroid transmission by insects, parasitic plants, and goats is summarized in Table 2.

ACFSVd was transmitted by two species of aphids and by the larvae of a codling moth that had been feeding directly on symptomatic, viroid-infected apple trees [10]. Desvignes [72] reported that *Myzus persicae* (the green peach aphid) experimentally transmitted PLMVd, but at a low rate, suggesting minor relevance under natural peach-orchard conditions [82]. PSTVd was transmitted by the aphid *Macrosiphum euphorbiae* in a non-persistent manner [73], and persistently by the green peach aphid when the viroid was encapsidated in potato-leafroll-virus (PLRV) particles [74,75,76]. The encapsidated viroid was transmitted to potato, *Physalis floridana*, and *Datura stramonium*, although the aphid species was not a significant vector for transmitting PSTVd alone. In potato, the efficiency of the green-peach-aphid transmission of PSTVd when co-transmitted with PLRV ranged from 0% to 55% depending on the potato cultivar used as the viroid inoculum or test plant [83]. TPMVd was reported to be efficiently transmitted by the green peach aphid [77]. However, the possibility of transmission of a pospiviroid by this aphid in the absence of a helper virus is currently suggested to be discounted [84]. Bumblebees have been shown to efficiently transmit TASVd [64] and TCDVd [78] in tomato plants. TASVd in the Netherlands is the most prevalent pospiviroid in ornamentals, from which it may cause outbreaks in tomatoes [85]. ASSVd is transmitted by the greenhouse whitefly from ASSVd-infected herbaceous hosts such as bean and cucumber to cucumber, bean, tomato and pea plants [79]. The transmission to cucumber was enhanced by cucumber-phloem protein 2, which forms a stable protein/ASSVd RNA complex in infected plants [79]. The hemi-parasitic plant mistletoe is frequently found in apple orchards. Mistletoe comes directly into contact with apple trees through haustoria, during which it may become systemically infected with ACFSVd and transmit the viroid from infected to uninfected trees [10]. Domesticated goats have been reported to transmit viroids when their horns were rubbed against viroid-infected trees [80,81]. Transmission via goats could have facilitated the long-range spread of viroids among cultivated and wild plants and vice versa, as well as among graft-incompatible plants. 

### 5.2. Fungal Transmission by Phytopathogenic Ascomycetes

ASBVd, of the family *Avsunviroidae*, has been reported to replicate in non-plant hosts: the unicellular yeast fungus *Saccharomyces cerevisiae* [86] and the filamentous blue-green cyanobacterium *Nostoc* sp. PCC 7120 [87]. Recently, it has been demonstrated that viroids infect and replicate in phytopathogenic ascomycete fungi [12]). In this study, full-length monomeric cDNA clones of seven viroids (Table 3), were tested for their infectivity to three fungi, *Cryphonectria parasitica*, *Valsa mali*, and *Fusarium graminearum* using the transfection of fungal spheroplasts. Among 21 viroid–fungus combinations, six (IrVd-1+*C. parasitica*, HSVd+*C. parasitica*/*V. mali*/*F. graminearum*, ASBVd+*C. parasitica*/*V. mali*) showed stable viroid accumulation in the fungi, persisting for at least eight subcultures, although its accumulation level was at much lower level relative to that in a plant host. Moreover, the viroids were horizontally transmitted during hyphal fusion and vertically maintained through conidial transfer, indicating that these phytopathogenic ascomycete fungi can support viroid replication. Most viroid infections were asymptomatic in the fungi, but HSVd infection significantly reduced the growth and virulence of *V. mali*. It has been suggested that viroid replication in fungi should also be verified by other detection techniques [88]. The sequence analysis of the HSVd, which accumulated in *F. graminearum*, and of the ASBVd, which accumulated in *C. parasitica*, after eight fungal subcultures showed nucleotide-sequence substitutions, suggesting that these viroids were replicating in and adapting to the fungal hosts [89]. Nucleotide-sequence substitutions were single-site substitutions, except for two ASBVd mutants, with interchanges occurring between adenine/guanine or cytosine/uracil. In addition, the sequence junction of the circularized plus RNAs of HSVd, which accumulated in *F*. *graminearum*, was determined by inverse RT-PCR in order to provide additional supporting evidence for viroid replication. This additional supporting evidence indicates that the viroid replicated and adapted in fungi and suggests that genome evolution or adaptation may occur during viroid replication in fungi [89].

When HSVd-infected *F. graminearum* was inoculated to *Nicotiana benthamiana*, the plants became systemically infected with the viroid seven days later. Conversely, when viroid-free *F. graminearum* was inoculated to HSVd-infected *N. benthamiana*, the fungus acquired HSVd from plants and became viroid-infected as shown by re-isolating the fungus from plants [12]. This finding demonstrates a two-way horizontal transfer of viroid between plant and fungus. Notably, such a bidirectional transfer between plants and pathogenic fungi has also been demonstrated in plants with the following viruses: cucumber mosaic virus, tobacco mosaic virus, and Cryphonectria hypovirus 1 [90,91]. 

As plants commonly host various fungi in nature [92], the transfer of parasitic molecules/organisms between plants and fungi as they exchange water, nutrients, and effector proteins may occur during colonization of a plant by a fungus. Thus, fungi may be previously under-estimated biological vectors of viroids and plant viruses in nature. Future studies of viroid epidemiology should include the possible role of fungi in their spread, since fungi have been reported to take up small RNA molecules from plant cells, as well as transfer small fungal RNAs to plants in order to regulate host immunity responses [93,94]. Our understanding of the viroid/virus cross-kingdom between plants and fungi is still in its infancy. Further studies to decipher this novel phenomenon of viroid transmission to fungi are necessary.

**Table 3:** Vector Transmission of Viroids by Infectious cDNA Clones.

Viroid Acronym	Infectious cDNA Clones	Reference
AHVd	Dimeric head-to-tail transcripts of AHVd RNA are infectious when inoculated to apple seedlings, thus demonstrating that this RNA is a viroid.	[95,96]
ASBVd ASSVd CSVd HSVd IrVd PLMVd PSTVd	Monomeric full-length RNA transcripts of seven viroid cDNA clones were inoculated by transfection to spheroplasts of three plant-pathogenic ascomycetes filamentous fungi, namely *Cryphonectria parasitica*, *Valsa mali* and *Fusarium graminearun*. Transmitted HSVd, IrVd and ASBVd can stably replicate in at least one of those fungi. The other five viroids stopped replication after the eighth subculture of the transfected fungi.	[12]
ASSVd	Infection of apple and pear seedlings by agroinfection of ASSVd recombinant constructs of ASSVd	[97]
ASSVd	Infection of nine herbaceous plant species by mechanical inoculation of in vitro ASSVd dimeric transcripts and to a lesser degree by dimeric DNA plasmids or sap inoculation	[98]
CbVds	RNAs synthesized in vitro from infecious clones of CbVd-1, -3, -5, and -6 were used as inocula on healthy coleus to study the biological properties of the four viroids. The first detection time for the four CbVds was different, ranging from about a month and a half to 10 months.	[48]
CbVd-7	To study the infectivity of the novel CbVd-7, RNA transcribed from CbVd-7 clones containing either monomeric, dimeric, or trimeric CbVd-7 sequences was used as inoculum to infect healthy coleus plants. Transcribed RNA was infectious.	[11]
CCCVd	A cDNA clone of the oil-palm variant CCCVd 246op was mechanically transmitted to oil-palm seedlings. Orange-spotting symptoms were observed within six months of inoculation, confirming the pathogenicity of CCCVd246op	[99]
CChMVd	CChMVd was transmitted by plasmids containing the viroid dimeric head-to-tail cDNA inserts or their in vitro transcripts.	[100]
CSVd	In vitro-transcribed CSVd was infectious to the chrysanthemum and other plants, Thus extending the CSVd host range and its potoential to spread the disease	[101]
GLVd	Healthy grapevine seedlings were infected following slash inoculation with in vitro transcripts of GLVd	[57,95]
PLMVd	PLMVd was transmitted by the viroid cDNA clones/in vitro transcripts	[41,102,103]
PoLVd	Mechanical inoculation by leaf rubbing of head-to-tail dimeric transcripts of PoLVd generated in vitro was successful as two out of six Portulaca plants became infected.	[104]
PSTVd, CSVd TCDVd, HSVd	Infectious monomeric linear viroid RNA from a cDNA clone will facilitate mutational analyses by in vitro mutagenesis	[105]
CEVd	The nucleotide sequence of full-length cDNA clones of CEVdc purified from citron showed exchanges that have not been reported for other CEVd variants	[106]

## 6. Mycoviruses (Fungal Viruses) and Possible Existence of Mycoviroids (Fungal Vroids)

Mycoviruses (fungal viruses) are viruses that infect and replicate in fungi and are usually associated with symptomless infections [107]. These viruses are widespread in all major taxa of fungi, have shown remarkable diversity, and some may induce a reduction in the virulence of phytopathogenic fungi and may be used in their biological control, such as the mycoviruses of chestnut-blight fungus *Cryphonectria parasitica* [107]. It is worth noting that the first mycovirus was reported in cultivated mushroom, *Agaricus bisporus*, a basidiomycete, in 1962 [108] and there are now over 90 fungal viruses belonging to 10 families [107]. Infection and replication of viroids in fungi under laboratory conditions [12] may suggest that mycoviroids (fungal viroids) exist in nature. A mycoviroid may be defined as a viroid that has the ability to infect healthy fungi. Wei et al. [12] reported that HSVd infection significantly reduced the growth and virulence of the phytopathogenic fungal host *Valsa mali,* the causal agent of valsa canker in apple. Thus, this mycoviroid could conceivably be exploited for development of novel biocontrol strategies for *V. mali*. It is expected in the next several years that research on mycoviroids will be expanded into other host/viroid systems, which may include major taxa of fungi, oomycetes, and possibly the unculturable biotrophic fungi as well as many viroid species and their variants. 

## 7. Experimental Transmission by cDNA Clones

The transmission of viroids by infectious cDNA clones is summarized in Table 3. Three ascomycetous filamentous fungi, namely *C. parasitica*, *V. mali* and *F. graminearun*, which are the causal agents of chestnut blight, apple tree canker and wheat/barley head blight and maize ear rot diseases, respectively, were investigated for the possible replication of monomeric, full-length RNA transcripts of seven cDNA clones after inoculation of fungal spheroplasts [12]. ASSVd, CSVd, PLMVd and PSTVd initially replicated in the inoculated fungal hosts, then they were eliminated after the eighth fungal subculture; however, HSVd, IrVd-1 and ASBVd consistently replicated in at least one of those fungi [12]. HSVd replicated in the three fungi while ASBVd replicated in *C. parasitica* and *V. mali*, and IrVd-1 only replicated in *C. parasitica*. The growth of *V*. *mali* was severely affected by HSVd infection. The deletion of dicer-like genes from *C. parasitica* and *F. graminearun* caused a significant increase in the HSVd titer in these fungi [12]. Infectious cDNA clones were used as vectors for transmitting AHVd, ASSVd, CbVds, CbVd-7, CCCVd, CChMVd, GLVd, PLMVd, PoLVd, PSTVd, CSVd, TCDVd, HSVd, and CEVd in order to study the biological properties of the viroids of interest (Table 3).

## 8. Control of Viroid Transmission

The movement of viroid-infected, vegetatively propagated plant material has contributed significantly to the global spread of viroids. Viroids are considered to be of quarantine and certification importance in many countries and their regulations may vary from country to country or from continent to continent. Viroids of quarantine importance in the United States include: HSVd (cachexia strain), PBCVd, and PSTVd; in Canada: ASSVd, PBCVd and PSTVd; in Mexico: ChCMVd, CSVd, CEVd, CCCVd, ELVd, HSVd (cachexia strain), PLMVd, PBCVd and PSTVd [109]. The European Union is considered a unique “country”/region with different entry points. The inspection service at each entry point is responsible for preventing any circulation of infected vegetatively propagative plants. The following viroids must be absent from certified propagative material in the EU: ADFVd, ASSVd, CEVd, HSVd (cachexia strain), PBCVd, PLMVd [109] and CCCVd [110]. Other countries may have similar or different viroids of quarantine or certification importance. For example, Australia considers all viroids to be of quarantine importance; however, China considers the following viroids to be of quarantine significance: HSVd (cachexia strain), CEVd and CCCVd; while Chile considers ASBVd, CCCVd, PSTVd, TASVd and TCDVd to be of quarantine importance [109,111]. A complete list of viroids of quarantine importance in many countries was reported by [109]. Next-generation sequencing may be used as the major diagnostic method for viroids in quarantine and certification systems [111,112], where various restrictions apply and viroid detection and identification is critical.

Cultural practices such as de-leafing, fruit picking, other frequent hands-on activities, pruning, and long production cycles have created many opportunities for the mechanical transmission of viroids. Therefore, it is necessary to use effective disinfectants to prevent viroid dissemination. Several chemical disinfectants have been described which are effective in disinfecting cutting tools against viroid transmission [113]. Among these disinfectants, household bleach (0.5–1% sodium hypochlorite) diluted to 10–20% was effective against many contaminating viroids, thus preventing the mechanical transmission of viroids [113].

At least 18 viroids have been reported as seed-transmitted (Table 1). Therefore, it is important for planting seeds of interest to select seed lots that have been certified as viroid-free by a rigorous seed-certification program.

Different approaches have been utilized over the years for viroid elimination from infected plants [114,115,116]. Recently, Barba et al. [117] described the possible viroid elimination from infected plant tissue by thermotherapy, cold therapy, tissue culture, in vitro micrografting, or cryotherapy. Among the viroids that were eliminated from infected plants by one or more of these techniques were ASSVd from apple and pear, HLVd and HSVd from hop, CSVd, CChMVd, and HSVd from chrysanthemum, HSVd from peach and pear, PSTVd from potato and tomato, PLMVd from peach, CEVd and HSVd from citrus, and CEVd and TCDVd from tomato [117]. Viroid-elimination frequency varied with viroid–host combinations.

## Figures and Tables

**Table 1:** Seed and Pollen Transmission of Viroids.

Type of Transmission	Viroid Acronym	Reference
Seed	ACFSVd (in apple and mistletoe)	[10]
	ASBVd (in avocado)	[45]
	ASSVd (in apple)	[29,46]
	CbVd-1 (in coleus)	[47]
	CbVd-5 and CbVd-6	[48]
	CCCVd (in *Cocos nucifera*)	[49]
	CEVd (in *Impatients* and *Verbena* species)	[50]
	CLVd (in petunia and tomato)	[51]
	CSVd ( in chrysanthemum)	[52,53]
	CSVd (in tomato)	[54]
	ELVd (in eggplant)	[44,55]
	GYSVd-1 (in grapevine)	[56,57]
	HLVd (in hops)	[58,59]
	HSVd (in grapevine)	[56]
	PCFVd (in pepper)	[60]
	PSTVd (in potato)	[61]
	PSTVd (in tomato and potato)	[62]
	PSTVd (in true seed)	[63]
	PSTVd (in tomato, pepper, petunia)	[51]
	TASVd (in tomato)	[64]
	TCDVd (in tomato)	[65,66]
	TCDVd (in petunia and tomato)	[51]
Pollen	ASBVd	[67]
	CCCVd (in *Cocos nucifera*)	[49]
	CSVd ( in chrysanthemum)	[52,53]
	CSVd (in tomato)	[54]
	HLVd (in hops)	[58,59]
	PLMVd (in peach)	[68]
	PSTVd (in potato)	[61,69]
	PSTVd (in tomato)	[70]

**Table 2:** Insects, Parasitic Plants and Goats Transmission of Viroids.

Vector	Viroid Acronym	Reference
**INSECTS**		
A-Aphids		
*Dysaphis plantaginea*	ACFSVd	[10]
*Myzus persicae*	ACFSVd	[10]
*M. persicae*	PLMVd	[72]
*Macrosiphum euphorbiae*	PSTVd	[73]
*M. persicae*	PSTVd	[74,75,76]
*M. persicae*	TPMVd	[77]
B-Codling Moth		
*Cydia pomonella*	ACFSVd	[10]
	C-Bumble Bees	
*Bombus ignitus*	TASVd	[64]
	TCDVd	[78]
	D-White flies	
*Trialeurodes vaporariorum*	ASSVd	[79]
**PARASITIC PLANTS**		
Mistletoe	ACFSVd	[10]
*Viscum album* subsp. *Album*		
**DOMESTICATED GOATS**		
Goat (*Capra hircus*)	CEVd, HSVd	[80]
horns	PLMVd	[81]

## Abbreviations

ACFSVd, apple chlorotic fruit spot viroid, ADFVd, apple dimple fruit viroid; AFCVd, apple fruit crinkle viroid; AHVd, apple hammerhead viroid; ASSVd, apple scar skin viroid; AGVd, Australian grapevine viroid; ASBVd, avocado sunblotch viroid; CChMVd, chrysanthemum chlorotic mottle viroid; CSVd, chrysanthemum stunt viroid; CBCVd, citrus bark cracking viroid; CBLVd; citrus bent leaf viroid; CDVd, citrus dwarfing viroid; CEVd, citrus exocortis viroid; CVd-V, citrus viroid V; CVd-VI, citrus viroid VI; CCCVd, coconut cadang-cadang viroid; CTiVd, coconut tinangaja viroid; CbVd-1, coleus blumei viroid 1; CbVd-3, coleus blumei viroid 3; CbVd-5, coleus blumei viroid 5; CbVd-6, coleus blumei viroid 6; CbVd-7, coleus blumei viroid 7; CLVd, columnea latent viroid; ELVd, eggplant latent viroid; GLVd; grapevine latent viroid; GYSVd 1, grapevine yellow speckle viroid-1; GYSVd 2, grapevine yellow speckle viroid-2; HLVd, hop latent viroid; HSVd, hop stunt viroid; IrVd-1, Iresine viroid 1; PCFVd, pepper chat fruit viroid; PLMVd, peach latent mosaic viroid; PBCVd, pear blister canker viroid; PoLVd, portulaca latent viroid; PSTVd, potato spindle tuber viroid; PVd 2, persimmon viroid-2; TASVd, tomato apical stunt viroid; TCDVd, tomato chlorotic dwarf viroid; TPMVd, tomato planta macho viroid.
